# Uni-hemispheric dual-site anodal tDCS (M1-DLPFC) for upper limb motor function and spasticity in chronic stroke: a randomized clinical trial

**DOI:** 10.3389/fneur.2025.1662978

**Published:** 2026-02-25

**Authors:** Kamran Ezzati, Sofia Naghdi, Iraj Abdollahi, Somaye Azarnia, Shapoor Jaberzade

**Affiliations:** 1 Department of Physiotherapy, Neuroscience Research Center, Trauma Institute, Poursina Hospital, School of Medicine, Guilan University of Medical Sciences, Rasht, Iran; 2 Department of Physiotherapy, Faculty of Rehabilitation, Tehran University of Medical Sciences, Tehran, Iran; 3 Department of Physiotherapy, Faculty of Rehabilitation, University of Social Welfare and Rehabilitation Sciences, Tehran, Iran; 4 Neuroscience Research Center, Physical Therapy Department, Faculty of Medicine, Guilan University of Medical Sciences, Rasht, Iran; 5 Department of Physiotherapy, Faculty of Medicine, Nursing and Health Sciences, Monash University, Melbourne, VIC, Australia

**Keywords:** chronic stroke, M1-DLPFC stimulation, non-invasive brain stimulation, spasticity, transcranial direct current stimulation, upper extremity function

## Abstract

**Background/objectives:**

Upper extremity impairment significantly affects motor function and quality of life after stroke. This study investigated the safety and efficacy of a non-invasive brain stimulation approach, uni-hemispheric concurrent dual-site anodal transcranial direct current stimulation (a-tDCS) targeting the primary motor cortex (M1) and dorsolateral prefrontal cortex (DLPFC), to improve upper extremity performance in chronic stroke.

**Methods:**

This double-blind, randomized, sham-controlled study involved 38 chronic stroke patients to evaluate the safety and efficacy of uni-hemispheric concurrent dual-site anodal transcranial direct current stimulation (a-tDCS). Participants were randomly assigned to one of two groups: experimental group 1 (a-tDCS at 2 mA targeting M1 and DLPFC concurrently) or experimental group 2 (active a-tDCS at 2 mA over M1 with sham stimulation over DLPFC), with each receiving 20-min sessions over five consecutive days. Upper extremity motor function (Fugl-Meyer Assessment—FMA) and spasticity (Modified Modified Ashworth Scale—MMAS) were assessed at baseline and 24 h following the final intervention. The procedure was deemed safe. Statistical analysis involved the U Mann–Whitney test for between-group comparisons and the Wilcoxon signed-rank test for within-group changes.

**Results:**

The results demonstrated that uni-hemispheric concurrent dual-site a-tDCS targeting M1 and DLPFC in experimental group 1 did not lead to statistically significant improvements in upper extremity motor function, elbow and wrist flexor spasticity, or range of motion in this cohort of chronic stroke patients. Furthermore, no statistically significant differences were found between experimental group 1 and experimental group 2 (the sham control group) for any of the measured outcomes (*p* ≥ 0.05).

**Conclusion:**

Uni-hemispheric concurrent dual-site a-tDCS targeting both M1 and DLPFC did not demonstrate a superior effect on upper extremity motor recovery compared to a-tDCS applied solely to M1 in chronic stroke patients.

## 1 Introduction

Stroke, a leading cause of long-term disability affecting a significant and growing population, often results in substantial and persistent impairments in upper extremity motor function, creating a considerable burden individuals and healthcare systems. While traditional rehabilitation methods form the cornerstone of recovery, a significant number of stroke survivors experience incomplete functional restoration, underscoring the critical need for innovative and more effective therapeutic interventions (1, 2). Transcranial direct current stimulation (tDCS) has emerged as a promising noninvasive technique, capable of influencing neuronal activity and promoting neuroplasticity, offering a potential avenue to enhance motor recovery in stroke patients (3, 4).

tDCS involves the application of weak, constant electrical currents to specific brain regions through electrodes placed on the scalp (5, 6). The polarity and precise parameters of stimulation are key determinants of its effects; generally, anodal stimulation is associated with facilitating excitatory plasticity by depolarizing resting membrane potentials, whereas cathodal stimulation induces inhibitory effects by hyperpolarizing neuronal networks (7). Compared to other neurostimulation modalities such as repetitive transcranial magnetic stimulation (rTMS), tDCS offers practical advantages, including its simplicity, portability, and lower cost, making it potentially more accessible for widespread clinical application (8, 9).

Recent research has increasingly focused on the potential benefits of simultaneous dual-site tDCS, particularly unihemispheric concurrent dual-site stimulation (UHCDS) targeting the primary motor cortex (M1) and the dorsolateral prefrontal cortex (DLPFC) within the ipsilateral hemisphere. M1, a critical area for motor execution, has long been a primary target for interventions aimed at motor recovery. However, there is a growing appreciation for the crucial role of the DLPFC in higher level cognitive functions that under-pin motor planning, learning, and cognitive-motor integration, suggesting it could be a valuable target for enhancing rehabilitation outcomes.

The theoretical basis for this dual-site approach lies in established principles of synaptic and structural neuroplasticity. Anodal tDCS applied to M1 has been shown to in-duce long-term potentiation (LTP)-like plasticity, characterized by the strengthening of synaptic connections and a sustained increase in corticospinal excitability (10, 11). Ex-tending this facilitatory approach to the DLPFC, an area integral to executive functions and motor intention, could theoretically enhance the integration of cognitive control and motor execution pathways, thereby augmenting the recovery process. This emphasis on the cognitive-motor interface aligns with evidence suggesting that active cognitive engagement during motor learning promotes better skill retention and generalization, key objectives in functional rehabilitation (12). While the application of tDCS to M1 is relatively well-studied, the optimal targets and stimulation parameters for effectively leveraging the interplay between motor and cognitive areas through dual-site stimulation remain an active area of investigation. Factors such as current density, electrode size, and stimulation duration exert complex and often non-linear effects on corticospinal excitability, highlighting the need for systematic research to define optimal protocols (13, 14).

This study aims to evaluate whether the combined application of a-tDCS to both M1 and DLPFC can result in superior improvements in motor function in individuals with chronic stroke.

## 2 Materials and methods

### 2.1 Participants and study design

This study employed a double-blind, randomized controlled trial design. A total of 38 chronic post-stroke patients (20 men, 18 women; mean age 64.34 years) were recruited and randomly assigned to one of two groups: an active dual-site tDCS group (“experi-mental1”) and an active M1/sham DLPFC tDCS control group (“experimental2”). Both groups received a total of five sessions of tDCS intervention. All participants had experienced a first ischemic stroke in the middle cerebral artery (MCA) territory more than 6 months prior to enrollment (mean onset duration 34.2 weeks). These participants were selected from a cohort of 430 individuals admitted to Pars Hospital with a diagnosis of stroke between June 20, 2022, and July 20, 2023. Stroke diagnosis was confirmed through clinical assessment and neuroimaging (CT or MRI).

Inclusion criteria were: A Modified Modified Ashworth Scale (MMAS) score of 1 or higher for wrist flexor spasticity, the ability to communicate verbally in Persian, and a Persian Mini-Mental State Examination (MMSE) score of 23 or higher. Exclusion criteria included severe cognitive or memory impairment, upper limb deformities that would interfere with motor assessment or tDCS electrode placement, a family history of chromic neurological disorders (e.g., epilepsy), and the use of medications known to affect cognition (e.g., benzodiazepines, anticholinergics).

Outcome measures were collected at baseline (before the first tDCS session) and 24 h after the fifth and final tDCS intervention session. The primary outcome measures were the Fugl-Meyer Assessment (FMA) for upper extremity motor function and the Modified Modified Ashworth Scale (MMAS) for spasticity. Passive range of motion (PROM) for the upper extremity was also measured. Written informed consent was obtained from each participant prior to enrollment. The clinical trial code is IRCT20211030052912N1.

### 2.2 Randomization

**Figure 1:**
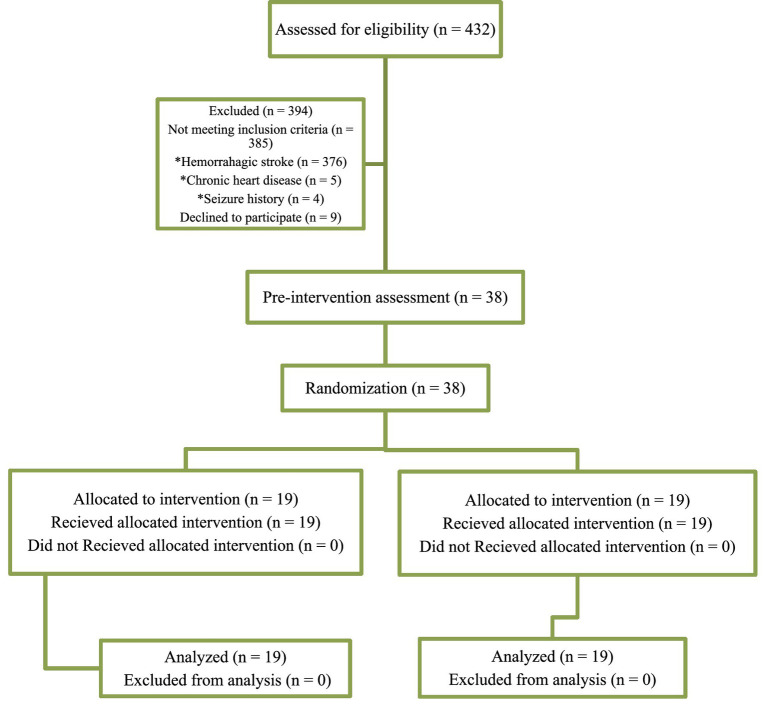
Consort diagram of patients.

Double-blind randomization was performed using the Randomization.com website. Participants were randomly allocated to one of the two experimental groups (1 and 2) using a computer-generated block randomization design. The allocation sequence was concealed from the researchers enrolling and assessing participants. Experiment 1 received five sessions of active anodal tDCS targeting M1 and DLPFC (a-tDCS M1-DLPFC). Experiment 2 received five sessions of active anodal tDCS targeting the M1 and sham stimulation targeting the DLPFC [a-tDCS M1-DLPFC (sham)]. The tDCS devices were programmed and operated by a researcher not involved in the participant recruitment or outcome assessment, ensuring blinding of both participants and assessors to the stimulation condition (Figure 1).

### 2.3 Transcranial direct current stimulation (tDCS)

**Figure 2:**
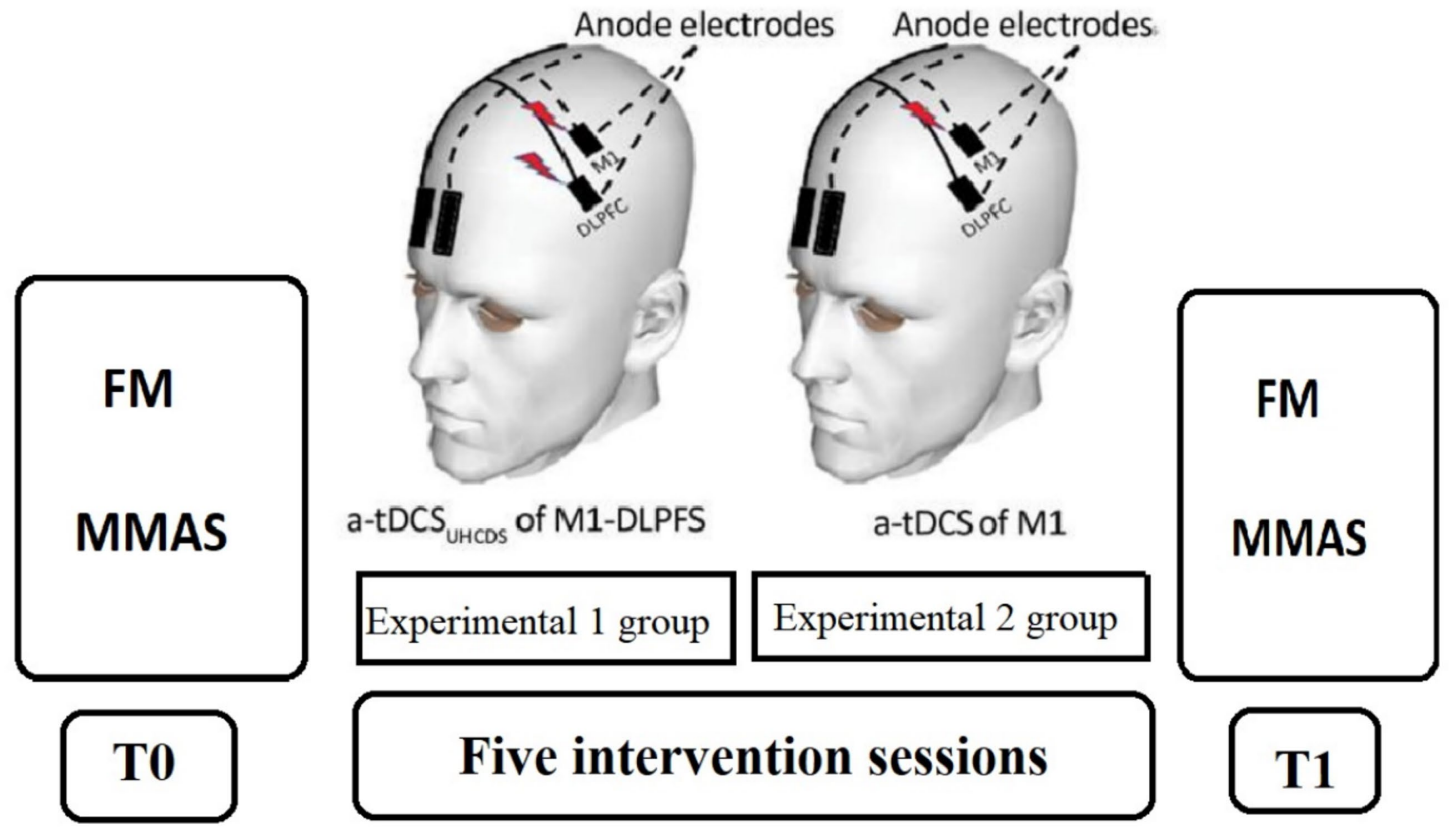
his figure is adapted from [the effects of anodal-tDCS on corticospinal excitability enhancement and its after-effects: conventional vs. unihemispheric concurrent dual-site stimulation, Vaseghi et al. (15)] schematic illustration of electrode montage in experimental 1: UHCDS a-tDCS (M1-DLPFC) and experimental 2: UHCDS a-tDCS (M1_real_-DLPFC _sham_); the reference electrodes were placed over the contralateral supraorbital area in two conditions. In both groups, the active electrodes were positioned over M1 and dorsolateral prefrontal cortex (DLPFC).

tDCS was delivered using two single-channel devices. Direct current was administered through saline-soaked electrodes. Electrode placement followed the international 10–20 EEG system. Active electrodes (4 × 4 cm2) were placed over the primary motor cortex (M1, C3/C4) and the dorsolateral prefrontal cortex (DLPFC, F3/F4) on the affected hemi-sphere. Reference electrodes (standard 5 × 7 cm2) were positioned on the contralateral supraorbital area (Figure 2) (15). A constant current of 1 mA was delivered for 20 min in both groups (10). However, in the “experimental2” group, DLPFC stimulation was turned off after 30 s to create the sham condition for this area (16) (Figure 2).

### 2.4 Outcome measures

Outcome measurements were collected before the first tDCS intervention and 24 h after the fifth intervention. The primary outcomes were FMA for upper extremity motor function and MMAS for spasticity, which were assessed through questionnaire and therapist evaluation. PROM for the upper extremity was also assessed.

### 2.5 Fugl-Meyer assessment (FMA)

The Fugl-Meyer Assessment is a widely used test to evaluate motor function in stroke patients, encompassing five domains: Motor function, Sensory function, Balance, Joint function, and Joint pain (17, 18). In this study, only the motor function domain of the up-per extremity was assessed. This domain evaluates upper limb tasks without compensation and voluntary joint movement. Each item within this domain is scored between 0 (inability) to 2 (full performance). The patient’s upper extremity was positioned appropriately for each test item.

### 2.6 Modified Modified Ashworth scale for spasticity (MMAS)

The Modified Modified Ashworth Scale (MMAS) is a clinical measure assessing muscle spasticity based on the resistance felt during passive movement (19). Spasticity is graded on a scale of 0 to 4, with higher scores indicating greater resistance. For this study, elbow flexor spasticity was measured. The patient was positioned supine with the head neutral and the arm at the side. The forearm was moved from maximum elbow flexion to full extension, and the level of resistance was recorded. One measurement was taken per session, with a maximum score of 4 representing severe spasticity and 0 indicating no spasticity (20). This assessment was conducted before the first treatment and after the fifth treatment to evaluate the intervention’s effect on spasticity.

### 2.7 Data analysis

Data analysis was performed using SPSS software (version 26, IBM SPSS Statistics for Windows, IBM Corp, Armonk, NY, USA). Continuous variables were presented as mean ± standard deviation. The Shapiro–Wilk test confirmed the normal distribution of the data. Between-group comparisons were conducted using the Mann–Whitney U test, and with-in-group comparisons were performed using the Wilcoxon signed-rank test. The level of statistical significance was set at *p* < 0.05. The sample size was determined using G*Power software (v3.1, Heinrich-Heine-University), based on the effect size (d = 2.0) from the Rayen study, a power of 0.90, and an alpha level (α) of 0.05. A 20% increase was added to account for potential attrition.

## 3 Results

### 3.1 Comparison of baseline characteristics and study measures

There were no significant differences in demographics, comorbidities, or spasticity levels between the two study groups (Table 1), indicating that the randomization process was effective in creating comparable groups at baseline. Several key outcomes were assessed in this study, including mean Ashworth scores for the elbow and wrist, FMA scores for UE, wrist, hand, and total performance and PROM scores for the UE. These were measured at baseline and after the intervention. Table 1 shows the demographic and clinical characteristics of the participants. Slightly more than half of the patients (approximately 50%) were female, with a mean age of 64.34 years (SD = 8.79) and a mean time since stroke onset of 32.82 weeks (SD = 6.99). As shown in Table 1, no significant differences in baseline characteristics were observed between the sham tDCS group and the active tDCS group (Table 1).

**Table 1:** Baseline characteristics of the study participants.

Characteristic		Study group						
		Sham tDCS (*n* = 19)		Active tDCS (*n* = 19)		Total (*n* = 38)		*p*-value
		Count	*N* %	Count	*N* %	Count	*N* %	
Gender	Female	8	42.11	12	63.16	20	52.63	0.194^*^
	Male	11	57.89	7	36.84	18	47.37	
Age	Mean (SD)	64.74 (7.59)		63.95 (10.04)		64.34 (8.79)		0.786^†^
	Range	(48.0, 75.0)		(51.0, 81.0)		(48.0, 81.0)		
Weeks since stroke	Mean (SD)	31.79 (4.71)		33.84 (8.73)		32.82 (6.99)		0.373^†^
	Range	(25.0, 40.0)		(24.0, 60.0)		(24.0, 60.0)		

* Pearson Chi-square tests.

† Independent T-test.

### 3.2 Comparison of Ashworth score of elbow and wrist before and after intervention in experimental groups

**Figure 3:**
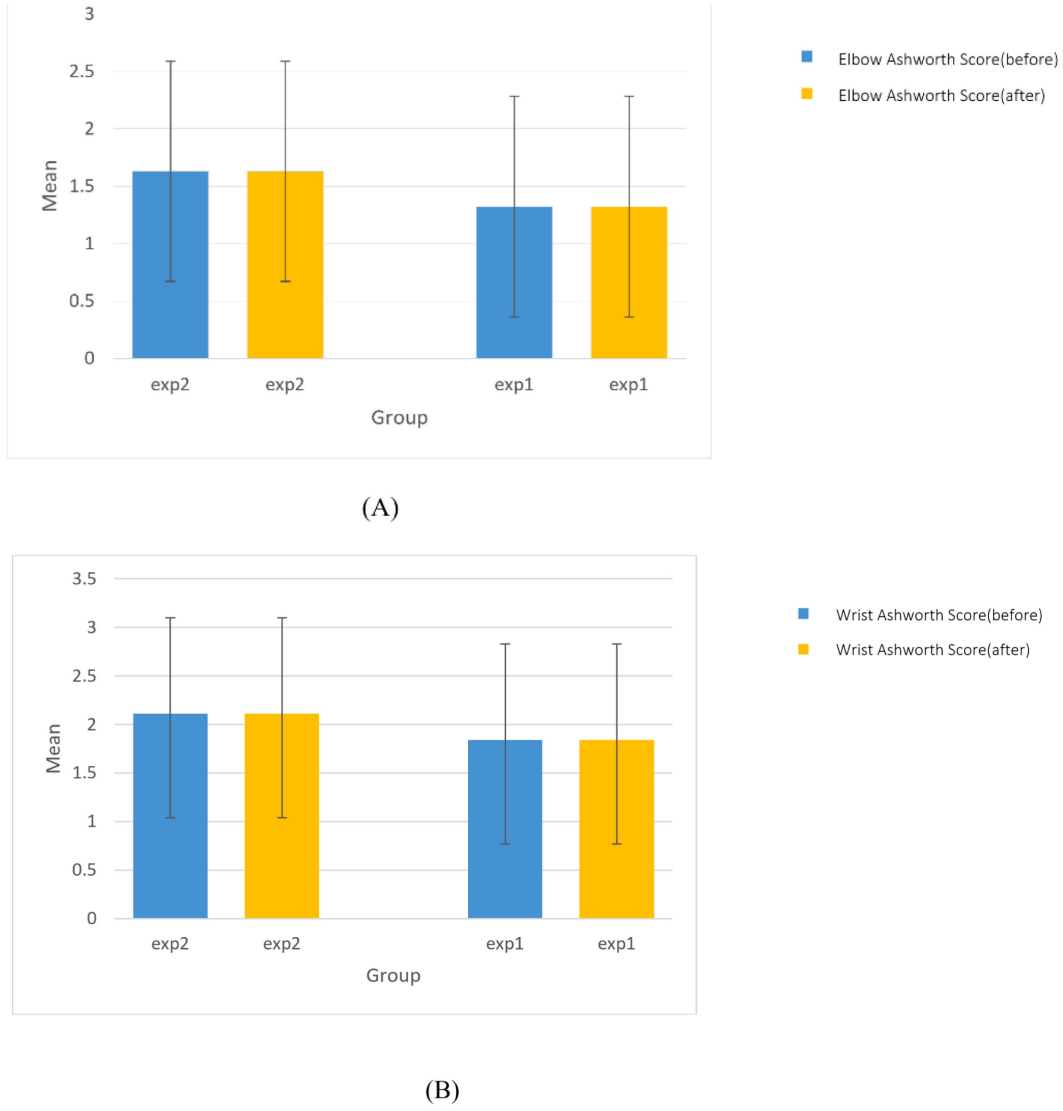
The comparison of **(A)** Ashworth score of elbow and **(B)** Ashworth score of wrist before and after the intervention between and within groups.

Figure 3 shows the Ashworth scores for the elbow and wrist before and after the intervention in both groups. At baseline, there were no significant differences between the groups in elbow spasticity (*p* = 0.999) or wrist spasticity (*p* = 0.999). Furthermore, no significant changes were observed within either group after the intervention for elbow or wrist spasticity. Between-group comparisons post-intervention also revealed no significant differences in elbow (*p* = 0.418) or wrist spasticity (*p* = 0.325). These results suggest that the a-tDCS intervention, regardless of the DLPFC stimulation condition, did not significantly impact elbow or wrist spasticity in this cohort (Figure 3).

### 3.3 Comparison of FMA score before and after intervention in experimental groups

**Figure 4:**
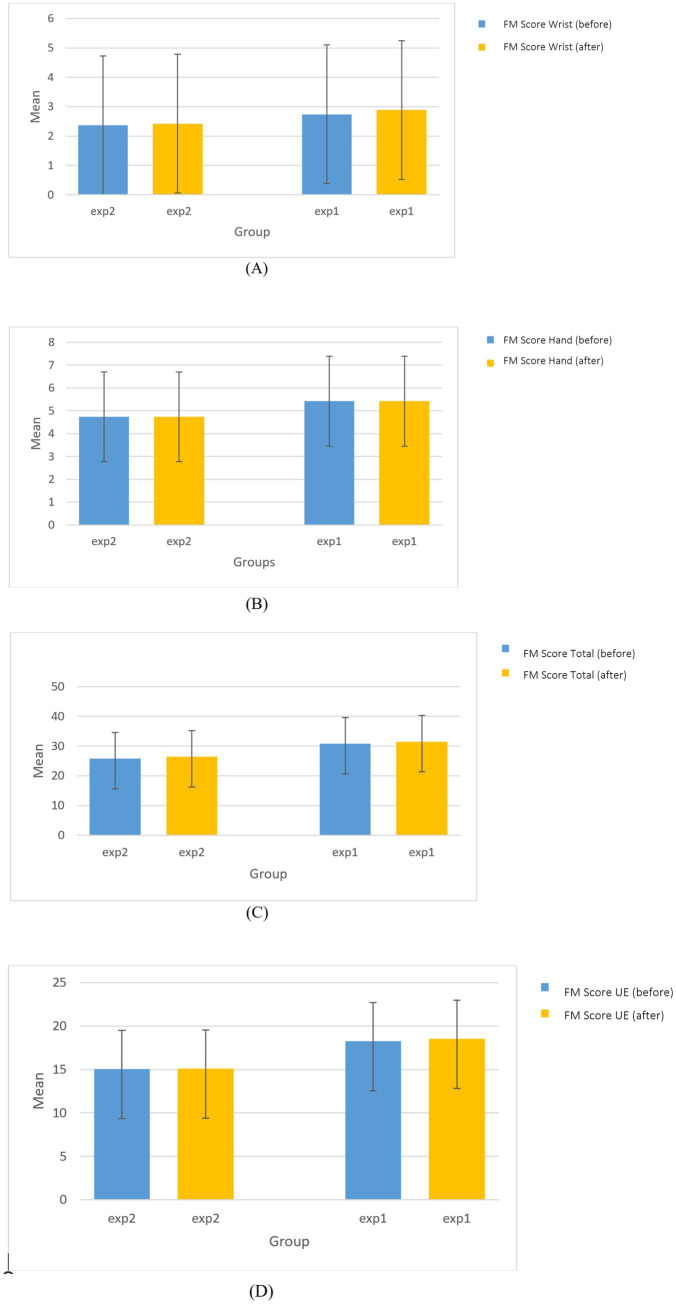
The comparison of FM score of **(A)** upper, **(B)** wrist, **(C)** hand, and **(D)** total before and after the intervention between and within groups.

The Fugl-Meyer Assessment of the upper extremity (Figure 4A) showed no statistically significant difference between the sham and active tDCS groups either before (*p* = 0.146) or after (*p* = 0.154) the intervention. In addition, FM score of wrist and hand did not show any significant difference between groups before the intervention (Wrist: *p* = 0.603, Hand: *p* = 0.296) or after the intervention (Wrist: *p* = 0.506, Hand: *p* = 0.296), and no significant changes were observed within groups (Wrist: Sham *p* = 0.317, Active *p* = 0.180; Hand: Sham *p* = 0.999, Active *p* = 0.999) (Figures 4B,C). Although the between group difference in the FMA total score was not significant at either time point (before: *p* = 0.111, after: *p* = 0.110), a statistically significant improvement within groups was observed in both the sham (*p* = 0.002) and active (*p* = 0.011) tDCS groups. This indicates that both the active and sham a-tDCS protocols were associated with improvements in overall upper extremity motor function, although there was no significant difference in the magnitude of improvement between the groups (Figure 4).

### 3.4 Comparison of PROM score of upper extremity before and after intervention in experimental groups

**Figure 5:**
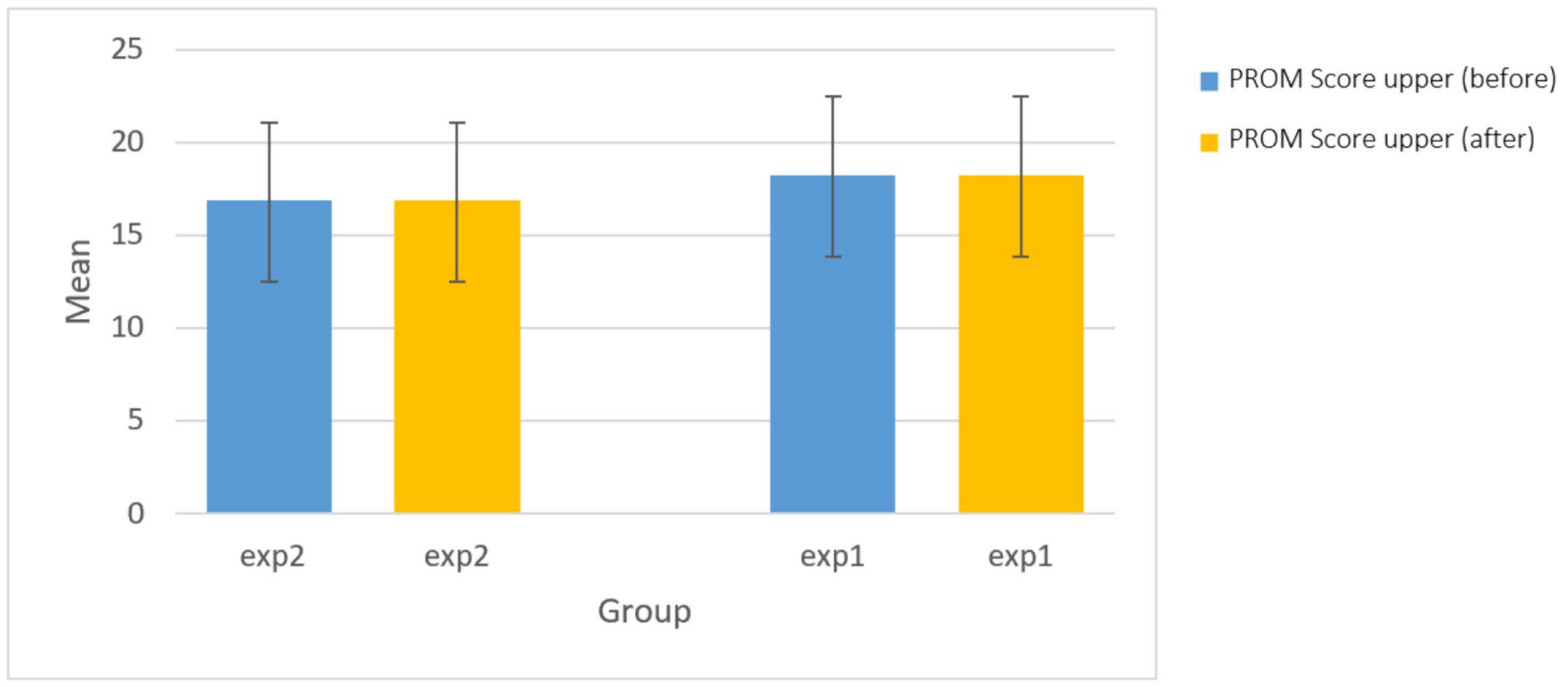
The comparison of passive range of motion (PROM) score of upper before and after intervention in experimental groups.

As shown in Figure 5, there were no statistically significant differences in the passive range of motion of the upper extremity between the sham and active tDCS groups before (*p* = 0.334) or after (*p* = 0.334) the intervention. This suggests that the a-tDCS intervention did not have a differential effect on upper extremity PROM between the two groups (Figure 5).

### 3.5 Comparison of joint pain score of upper before and after intervention in experimental group

**Figure 6:**
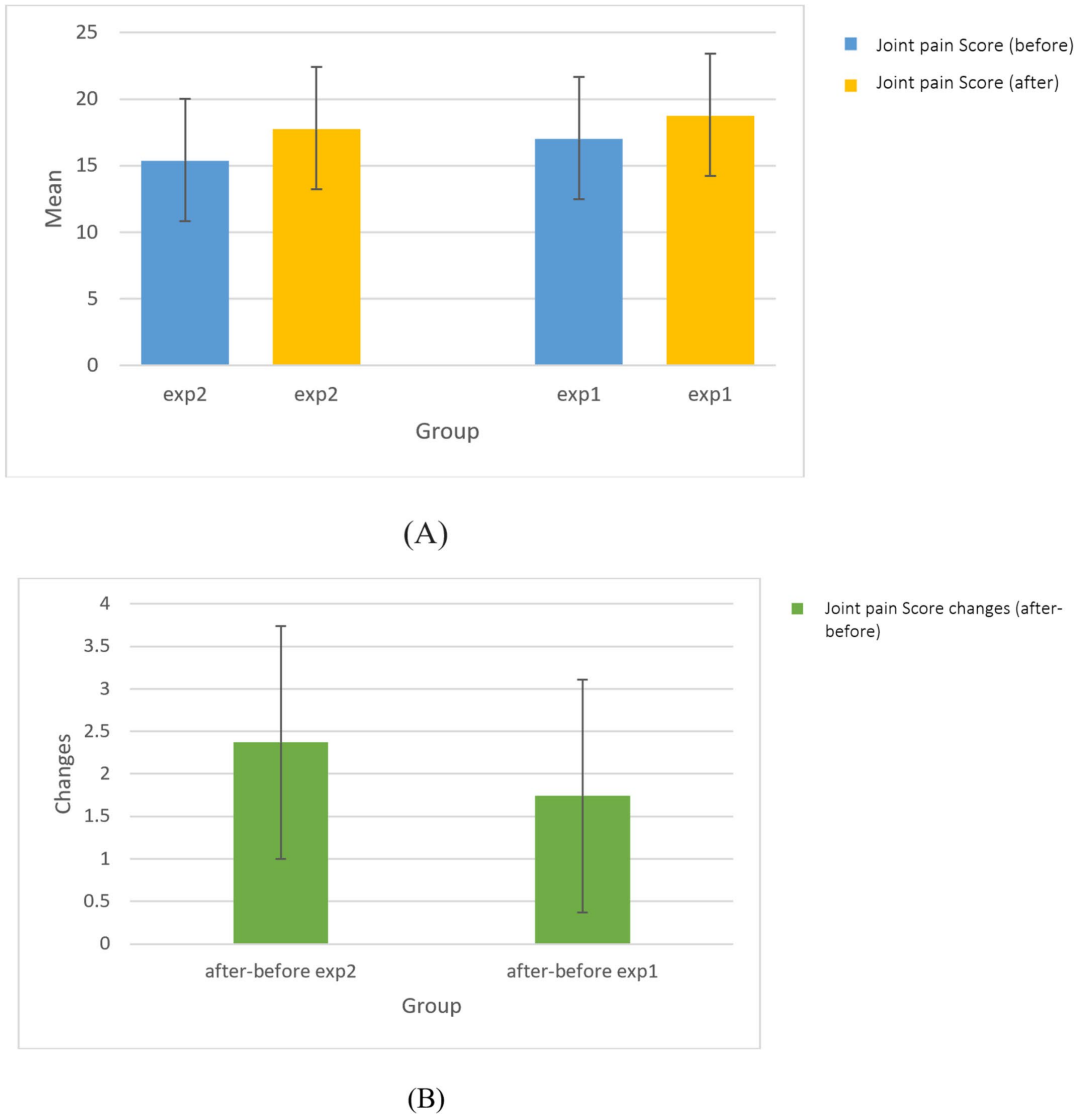
The comparison of **(A)** joint pain score changes and **(B)** upper before and after intervention in experimental groups.

Changes in joint pain scores between the experimental groups were statistically in-significant at baseline (*p* = 0.277), post-intervention (*p* = 0.339), and for the change score (*p* = 0.708). However, statistically significant reductions in joint pain within both the sham (*p* = 0.001) and active (*p* = 0.001) tDCS groups were observed (Figure 6). This indicates that both the active and sham tDCS protocols were associated with a reduction in reported joint pain.

## 4 Discussion

Our working hypothesis was that the two-point a-tDCS protocol (M1-DLPFC) would lead to significantly greater improvements in upper limb motor function compared to the single-point (M1 active—DLPFC sham) protocol in patients with chronic stroke. However, our results indicated that there was no statistically significant difference in FM scores be-tween the two-point and single-point stimulation protocols (Experiment 1 vs. Experiment 2). These results are consistent with the study by Achacheluee et al. who investigated the effects of a single session of UHCDS a-tDCS (M1-DLPFC) on upper limb motor function in patients with subacute stroke (21). Supporting this behavioral observation, Azarnia et al.’s randomized clinical trial in chronic stroke patients investigated the impact of uni-hemispheric dual-site anodal tDCS on brain metabolite changes using MRS. Their key finding was that there were no significant differences in NAA or Cho levels in the M1 region between the group receiving active M1 and sham DLPFC stimulation and the group receiving active stimulation to both sites. This suggests that, at a neurochemical level, the addition of DLPFC stimulation to M1 stimulation may not induce significantly different effects in the chronic stage (22).

Several studies have combined tDCS with physical therapy approaches, such as robotic-assisted therapy (23–26). Hesse et al. investigated the synergistic effects of transcranial direct current stimulation (tDCS) and a robotic hand device in patients with subacute stroke (23). Although improvements in FM scores were observed in both the experimental and sham groups, no significant difference was found between them. This lack of differential effect may be attributed to the specific stimulation parameters utilized or the neuro-plasticity mechanisms being targeted. In contrast, Edwards et al. demonstrated that the application of tDCS prior to robotic therapy improved motor function in patients with chronic stroke. The authors emphasized the importance of motor learning, which involves repetitive practice and is associated with long-term neural plasticity (26).

Other research has shown that the combination of tDCS with additional therapies, such as modified constraint-induced movement therapy (mCIMT), can have a positive effect on brain function (24). Motor function can be improved through robotic rehabilitation or virtual reality training. These findings highlight the need to integrate multisensory approaches to stimulate intact neural pathways for compensatory recovery. Intensive sensory input and motor training protocols are thought to promote neural circuit reorganization, particularly by activating underutilized pathways to compensate for damaged ones (27). These pathways may be effectively stimulated by rehabilitation strategies using robotics, virtual reality, and transcranial electrical stimulation.

In our study, joint pain showed significant improvement in both groups, consistent with the findings of Adams et al. who proposed tDCS as a potential stand-alone or ad-junctive therapy for the treatment of chronic pain, including knee and lower back pain (28). Importantly, a clinically meaningful reduction in pain (≥3 points) was observed in both groups and was maintained at follow-up, with no significant differences between the groups, similar to a randomized clinical trial in post-stroke shoulder pain (29). Baik et al. also observed significant within-group changes in pain scores following tDCS interventions (30). This suggests a potential analgesic effect of tDCS in chronic stroke, regardless of the specific stimulation montage used in this study.

Our results showed that UHCDS a-tDCS (M1-DLPFC) was not effective in reducing wrist and elbow flexor spasticity in patients with chronic stroke. These findings are in-consistent with studies by Viana et al. (31) and Grecco et al. Grecco et al. reported that tDCS combined with treadmill exercise enhanced motor training effects in children with spastic diplegic cerebral palsy (CP), likely by increasing cortical excitability and activating corticospinal pathways. Such facilitation may improve motor control in pediatric CP patients (32). Although Viana et al. observed a reduction in wrist spasticity when tDCS was used in conjunction with virtual reality therapy. In addition, a systematic review concluded that tDCS alone may not significantly reduce spasticity (33). These discrepancies highlight the importance of multimodal approaches and the potential limitations of isolated electrical stimulation in the treatment of spasticity in chronic stroke.

### 4.1 Limitations and suggestions for future research

This study has several limitations that may affect the interpretation and generalizability of its findings. The small sample size and limited number of treatment sessions (five) may limit the robustness of the conclusions, particularly regarding insignificant findings or the effect of lesion sites on motor recovery. Future studies with larger sample sizes, control groups, longer treatment durations, and neurophysiological assessments are recommended to address these limitations and provide a more comprehensive understanding of time-dependent changes following a-tDCS intervention. Future research could explore the optimal parameters for dual site a-tDCS in chronic stroke, including different stimulation intensities, durations, and combinations with various rehabilitation therapies, with a clear emphasis on the integration of motor practice protocols. Investigating the influence of individual lesion characteristics on the response to different tDCS protocols would also be valuable.

## 5 Conclusion

This study investigated the effects of unihemispheric simultaneous dual-site a-tDCS (a-tDCS-UHCDS) targeting M1 and dorsolateral prefrontal cortex (DLPFC) on upper limb motor function in chronic stroke patients. The results indicated that a-tDCS (M1-DLPFC) did not result in significantly greater improvements in upper limb motor recovery or a reduction in spasticity compared to a-tDCS [M1(real)—DLPFC(sham)]. However, both active and sham a-tDCS protocols led to an alleviation of joint pain. Despite the lack of a significant difference between the stimulation groups for motor outcomes, UHCDS a-tDCS may still have potential as a complementary treatment modality to improve motor function in this population, and further investigation with optimized parameters, larger samples, and integration with motor training is warranted.

## Data Availability

The raw data supporting the conclusions of this article will be made available by the authors, without undue reservation.

## References

[1] Pascual-Leone A Walsh V Rothwell J. Transcranial magnetic stimulation in cognitive neuroscience–virtual lesion, chronometry, and functional connectivity. Curr Opin Neurobiol. (2000) 10:232–7. doi: 10.1016/S0959-4388(00)00081-7, 10753803

[2] Antal A Nitsche MA Paulus W. Transcranial magnetic and direct current stimulation of the visual cortex. Suppl Clin Neurophysiol. (2003) 56:291–304. doi: 10.1016/S1567-424X(09)70233-8 14677406

[3] Kwakkel G Kollen BJ van der Grond J Prevo AJ. Probability of regaining dexterity in the flaccid upper limb: impact of severity of paresis and time since onset in acute stroke. Stroke. (2003) 34:2181–6. doi: 10.1161/01.STR.0000087172.16305.CD 12907818

[4] Nakayama H Jørgensen HS Raaschou HO Olsen TS. Recovery of upper extremity function in stroke patients: the Copenhagen stroke study. Arch Phys Med Rehabil. (1994) 75:394–8. doi: 10.1016/0003-9993(94)90161-9, 8172497

[5] Gomez Palacio Schjetnan A Faraji J Metz GA Tatsuno M Luczak A. Transcranial direct current stimulation in stroke rehabilitation: a review of recent advancements. Stroke Res Treat. (2013) 2013:170256. doi: 10.1155/2013/170256, 23533955 PMC3600193

[6] Nitsche MA Seeber A Frommann K Klein CC Rochford C Nitsche MS . Modulating parameters of excitability during and after transcranial direct current stimulation of the human motor cortex. J Physiol. (2005) 568:291–303. doi: 10.1113/jphysiol.2005.092429, 16002441 PMC1474757

[7] Nozari N Woodard K Thompson-Schill SL. Consequences of cathodal stimulation for behavior: when does it help and when does it hurt performance? PLoS One. (2014) 9:e84338. doi: 10.1371/journal.pone.0084338, 24409291 PMC3883650

[8] Utz KS Dimova V Oppenländer K Kerkhoff G. Electrified minds: transcranial direct current stimulation (tDCS) and galvanic vestibular stimulation (GVS) as methods of non-invasive brain stimulation in neuropsychology—a review of current data and future implications. Neuropsychologia. (2010) 48:2789–810. doi: 10.1016/j.neuropsychologia.2010.06.002, 20542047

[9] Gandiga PC Hummel FC Cohen LG. Transcranial DC stimulation (tDCS): a tool for double-blind sham-controlled clinical studies in brain stimulation. Clin Neurophysiol. (2006) 117:845–50. doi: 10.1016/j.clinph.2005.12.003, 16427357

[10] Hassanzahraee M Nitsche MA Zoghi M Jaberzadeh S. Determination of anodal tDCS intensity threshold for reversal of corticospinal excitability: an investigation for induction of counter-regulatory mechanisms. Sci Rep. (2020) 10:16108. doi: 10.1038/s41598-020-72909-4, 32999375 PMC7527486

[11] Samani MM Agboada D Jamil A Kuo M-F Nitsche MA. Titrating the neuroplastic effects of cathodal transcranial direct current stimulation (tDCS) over the primary motor cortex. Cortex. (2019) 119:350–61. doi: 10.1016/j.cortex.2019.04.016, 31195316

[12] Friedman NP Robbins TW. The role of prefrontal cortex in cognitive control and executive function. Neuropsychopharmacology. (2022) 47:72–89. doi: 10.1038/s41386-021-01132-0, 34408280 PMC8617292

[13] Bastani A Jaberzadeh S. A-tDCS differential modulation of corticospinal excitability: the effects of electrode size. Brain Stimul. (2013) 6:932–7. doi: 10.1016/j.brs.2013.04.005 23664681

[14] Nitsche MA Paulus W. Excitability changes induced in the human motor cortex by weak transcranial direct current stimulation. J Physiol. (2000) 527:633. doi: 10.1111/j.1469-7793.2000.t01-1-00633.x, 10990547 PMC2270099

[15] Vaseghi B Zoghi M Jaberzadeh S. How does anodal transcranial direct current stimulation of the pain neuromatrix affect brain excitability and pain perception? A randomised, double-blind, sham-control study. PLoS One. (2015) 10:e0118340. doi: 10.1371/journal.pone.0118340, 25738603 PMC4349802

[16] Bastani A Jaberzadeh S. Does anodal transcranial direct current stimulation enhance excitability of the motor cortex and motor function in healthy individuals and subjects with stroke: a systematic review and meta-analysis. Clin Neurophysiol. (2012) 123:644–57. doi: 10.1016/j.clinph.2011.08.029, 21978654

[17] Gladstone DJ Danells CJ Black SE. The Fugl-Meyer assessment of motor recovery after stroke: a critical review of its measurement properties. Neurorehabil Neural Repair. (2002) 16:232–40. doi: 10.1177/154596802401105171, 12234086

[18] Fugl-Meyer AR Jääskö L Leyman I Olsson S Steglind S. A method for evaluation of physical performance. Scand J Rehabil Med. (1975) 7:13–31. doi: 10.2340/1650197771331, 1135616

[19] Ansari NN Naghdi S Hasson S Fakhari Z Mashayekhi M Herasi M. Assessing the reliability of the modified modified Ashworth scale between two physiotherapists in adult patients with hemiplegia. NeuroRehabilitation. (2009) 25:235–40. doi: 10.3233/NRE-2009-0520, 20037215

[20] Naghdi S Nakhostin Ansari N Azarnia S Kazemnejad A. Interrater reliability of the modified modified Ashworth scale (MMAS) for patients with wrist flexor muscle spasticity. Physiother Theory Pract. (2008) 24:372–9. doi: 10.1080/09593980802278959 18821443

[21] Achacheluee ST Rahnama L Karimi N Abdollahi I Arslan SA Jaberzadeh S. The effect of unihemispheric concurrent dual-site transcranial direct current stimulation of primary motor and dorsolateral prefrontal cortices on motor function in patients with sub-acute stroke. Front Hum Neurosci. (2018) 12:441. doi: 10.3389/fnhum.2018.00441, 30429782 PMC6220031

[22] Azarnia S Ezzati K Saberi A Naghdi S Abdollahi I Jaberzadeh S. The effect of Uni-hemispheric dual-site anodal tDCS on brain metabolic changes in stroke patients: a randomized clinical trial. Brain Sci. (2023) 13:1100. doi: 10.3390/brainsci13071100, 37509030 PMC10377241

[23] Hesse S Waldner A Mehrholz J Tomelleri C Pohl M Werner C. Combined transcranial direct current stimulation and robot-assisted arm training in subacute stroke patients: an exploratory, randomized multicenter trial. Neurorehabil Neural Repair. (2011) 25:838–46. doi: 10.1177/1545968311413906, 21825004

[24] Rocha S Silva E Foerster Á Wiesiolek C Chagas AP Machado G . The impact of transcranial direct current stimulation (tDCS) combined with modified constraint-induced movement therapy (mCIMT) on upper limb function in chronic stroke: a double-blind randomized controlled trial. Disabil Rehabil. (2016) 38:653–60. doi: 10.3109/09638288.2015.1055382, 26061222

[25] Triccas LT Burridge JH Hughes A Verheyden G Desikan M Rothwell J. A double-blinded randomised controlled trial exploring the effect of anodal transcranial direct current stimulation and uni-lateral robot therapy for the impaired upper limb in sub-acute and chronic stroke. NeuroRehabilitation. (2015) 37:181–91. doi: 10.3233/NRE-151251, 26484510

[26] Edwards DJ Cortes M Rykman-Peltz A Chang J Elder J Thickbroom G . Clinical improvement with intensive robot-assisted arm training in chronic stroke is unchanged by supplementary tDCS. Restor Neurol Neurosci. (2019) 37:167–80. doi: 10.3233/RNN-180869, 30932903

[27] Poli P Morone G Rosati G Masiero S. Robotic technologies and rehabilitation: new tools for stroke patients’ therapy. Biomed Res Int. (2013) 2013:153872. doi: 10.1155/2013/153872, 24350244 PMC3852950

[28] Adams W Idnani S Kim J. Transcranial direct current stimulation for orthopedic pain: a systematic review with Meta-analysis. Brain Sci. (2024) 14:66. doi: 10.3390/brainsci14010066, 38248281 PMC10813248

[29] de Andressa Souza J Ferrari Corrêa JC Marduy A Dall'Agnol L de Gomes Sousa MH da Nunes Silva V . To combine or not to combine physical therapy with tDCS for stroke with shoulder pain? Analysis from a combination randomized clinical trial for rehabilitation of painful shoulder in stroke. Front Pain Res. (2021) 2:696547. doi: 10.3389/fpain.2021.696547, 35295490 PMC8915613

[30] Baik J-S Yang J-H Ko S-H Lee S-J Shin Y-I. Exploring the potential of transcranial direct current stimulation for relieving central post-stroke pain: a randomized controlled pilot study. Life. (2023) 13:1172. doi: 10.3390/life13051172, 37240817 PMC10221197

[31] Viana R Laurentino G Souza R Fonseca J Silva Filho E Dias S . Effects of the addition of transcranial direct current stimulation to virtual reality therapy after stroke: a pilot randomized controlled trial. NeuroRehabil. (2014) 34:437–46. doi: 10.3233/NRE-141065, 24473248

[32] Grecco LAC Duarte NdAC Mendonça ME Cimolin V Galli M Fregni F . Transcranial direct current stimulation during treadmill training in children with cerebral palsy: a randomized controlled double-blind clinical trial. Res Dev Disabil. (2014) 35:2840–8. doi: 10.1016/j.ridd.2014.07.030, 25105567

[33] Elsner B Kugler J Pohl M Mehrholz J. Transcranial direct current stimulation for improving spasticity after stroke: a systematic review with meta-analysis. J Rehabil Med. (2016) 48:565–70. doi: 10.2340/16501977-2097 27172484

